# Bone mineral density and microarchitecture improvement in a young patient with Hajdu-Cheney syndrome and autosomal dominant polycystic kidney disease treated with alendronate

**DOI:** 10.1016/j.bonr.2025.101838

**Published:** 2025-03-24

**Authors:** André Silva Franco, Valeria de Falco Caparbo, Elieser Hitoshi Watanabe, Rosa Maria Rodrigues Pereira, Luiz Fernando Onuchic

**Affiliations:** a Division of Rheumatology, Hospital das Clinicas HCFMUSP, Faculdade de Medicina da Universidade de Sao Paulo, SP, Brazil; b Divisions of Molecular Medicine and Nephrology, Faculdade de Medicina da Universidade de Sao Paulo, Sao Paulo, SP, Brazil

**Keywords:** Hajdu-Cheney syndrome, Autosomal dominant polycystic kidney disease, Osteoporosis, Fragility fracture, Bisphosphonate, HR-pQCT

## Abstract

**Introduction:**

Osteoporosis, typically seen in postmenopausal women, can also affect younger individuals, a condition known as Early-Onset Osteoporosis (EOOP). EOOP may be secondary to various conditions or arise from rare genetic disorders such as Hajdu-Cheney Syndrome (HCS), characterized by systemic bone involvement and fragility fractures.

**Case Report:**

A 14-year-old male presented with a distal left femur fragility fracture. His medical history included spina bifida and bilateral tarsal coalition, with no family history of osteoporosis, and polycystic kidneys associated with a positive family history of autosomal dominant polycystic kidney disease (ADPKD). Laboratory tests were unremarkable, but dual X-ray absorptiometry (DXA) revealed low bone mineral density (BMD), and high resolution peripheral quantitative computed tomography (HR-pQCT) showed decreased volumetric bone density (vBMD), particularly in the cortical bone. At age 17, his kidneys were cystic and mildly enlarged. Whole exome sequencing revealed a pathogenic variant in *NOTCH2*, confirming the diagnosis of HCS, and a very likely causative variant in *PKD1*, supporting the diagnosis of ADPKD.

The treatment regimen included weekly alendronate, impact exercise, a calcium-rich diet, and vitamin D supplementation. After 3 years, follow-up DXA and HR-pQCT demonstrated significant improvements in BMD and vBMD, mainly in the cortical bone.

**Discussion:**

This case highlights the effectiveness of alendronate in managing osteoporosis in a patient with HCS and ADPKD, despite the current lack of strong supportive evidence. Long-term monitoring revealed substantial improvements in bone density and microarchitecture, underscoring the importance of early diagnosis and intervention for genetic causes of osteoporosis to prevent fracture-related morbidity.

## Highlights

• A 14-year-old male with Hajdu-Cheney Syndrome (HCS) had a fragility fracture and low BMD.
• Genetic testing identified a pathogenic NOTCH2 variant, confirming HCS diagnosis.
• Treatment with alendronate, exercise, calcium, and vitamin D improved BMD.
• HR-pQCT revealed significant cortical bone density improvement after 3 years.
• First long-term HCS bone mass follow-up using DXA and HR-pQCT.

## 1 Introduction

Osteoporosis, characterized by the deterioration of bone microstructure, low bone mineral density (BMD), and an increased risk of fractures, is most commonly observed in postmenopausal women and tends to worsen with age (Veronese et al., 2024). However, it can also arise from other conditions, including prolonged glucocorticoid use, malabsorptive disorders (e.g., bariatric surgery, celiac disease, short bowel syndrome), chronic alcoholism, chronic inflammatory diseases (e.g., rheumatoid arthritis), and endocrine disorders (e.g., hyperparathyroidism, hypogonadism) (Sakka, 2022). In contrast, early-onset osteoporosis (EOOP) affects younger individuals and may result from nutritional deficiencies, chronic illnesses, long-term medication use, or primary conditions associated with rare genetic variants (Costantini et al., 2022).

The occurrence of a fragility fracture during childhood is a concerning event. Identifying the underlying cause is crucial for selecting the most appropriate treatment, as there are numerous potential diagnoses with varying treatment options. The primary goal is to reduce the risk of future fractures. After ruling out secondary causes, particularly in the pediatric population, it is important to consider primary causes of osteoporosis (Formosa et al., 2024).

Hajdu-Cheney syndrome (HCS) is a rare autosomal dominant condition caused by a pathogenic variant in the *NOTCH2* gene. This disorder affects different tissues/organs, including the skeleton (Simpson et al., 2011). Typical manifestations include acro-osteolysis, EOOP, craniofacial dysmorphism, short stature, and polycystic kidneys. The disease may be diagnosed in childhood or early adulthood, often following unexpected fragility fractures. Although Hajdu-Cheney Syndrome (HCS) was first described in 1948, the identification of its associated *NOTCH2* gene mutation was only reported in the last decade. As a result, there are limited studies on specific therapies for osteoporosis and their effects on bone in HCS patients (Pittaway et al., 2018). In this context, we report the case of an adolescent diagnosed with HCS was treated with alendronate, with follow-up of the response using dual-energy X-ray absorptiometry (DXA) and high-resolution peripheral quantitative computed tomography (HR-pQCT). Interestingly, his genetic assessment also revealed the likely diagnosis of an additional monogenic disease: autosomal dominant polycystic kidney disease (ADPKD). In his case, ADPKD was inherited from his father and was likely the fundamental cause of his polycystic kidney phenotype.

## 2 Case presentation

A 14-year-old male presented to our rheumatology clinic following the diagnosis and surgical treatment of a distal left femur fracture caused by fragility. The patient's medical history included spina bifida at birth, shown to be associated with a normal karyotype at the time, and bilateral tarsal coalition diagnosed at age 9. Aside from these conditions, the patient was otherwise healthy, with normal neurocognitive and physical development. His father had autosomal dominant polycystic kidney disease (ADPKD), while no family history of osteoporosis or fracture was reported. Physical examination revealed retrognathia.

**Fig. 1:**
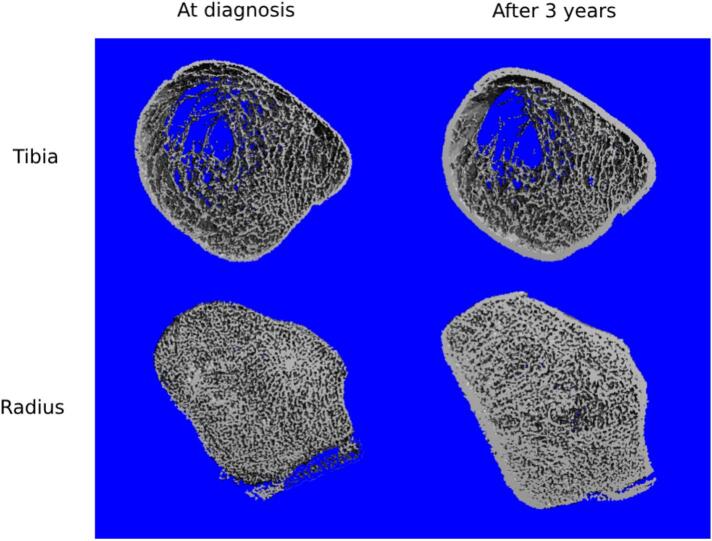
HR-pQCT at the diagnosis and after 3 years of treatment with alendronate.

Laboratory tests for osteoporosis revealed normal levels of serum calcium (9.6 mg/dL, reference value [RV]: 8.6–10.3), phosphorus (3.6 mg/dL, RV: 2.5–4.5), 25-OH-vitamin D (51 ng/mL, RV: > 20), PTH (43 pg/mL, RV: 19–64), TSH (1.2 mUI/mL, RV: 0,45–4.5), and free T4 (1.1 ng/dL, RV0.9–1.7). His renal function was preserved (serum creatinine 0.98 mg/dL, RV: 0.60–1.10; Schwartz-based estimated glomerular filtration rate: 72), 24-h urinary calcium was normal (90 mg, RV: 55 a 220 mg/24 h), inflammatory markers were within the normal range (C-reactive protein 0.03 mg/dL, RV < 0.5; erythrocyte sedimentation rate 6 mm, RV: 〈20), and screening for celiac disease was negative, effectively ruling out major causes of secondary osteoporosis. Renal ultrasound identified normal size kidneys with bilateral cortical renal cysts while at age 17 computed tomography showed mildly increased kidneys with multiple, bilateral cysts. Dual-energy X-ray absorptiometry (DXA) showed low bone mineral density (BMD) (Table 1). To further characterize bone microarchitecture, high-resolution peripheral quantitative computed tomography (HR-pQCT) was performed, revealing a reduction in volumetric bone density (vBMD). Despite the absence of reference values for pediatric patients, qualitative analysis of the image (Fig. 1) indicates significant impairment in the cortical compartment.

**Table 1:** DXA and HR-pQCT at the diagnosis and after 3 years of treatment with alendronate.

	At diagnosis		After 3 years of alendronate		Variation⁎	
HR-pQCT	Radius	Tibia	Radius	Tibia	Radius	Tibia
Tt.vBMD, mgHA/cm^3^	158	106	256	198	67.7 (+61.9 %)	91.5 (+85.9 %)
Tb.vBMD, mgHA/cm^3^	113	73	111	70	−2.5 (−2.2 %)	−2.8 (−3.9 %)
Ct.vBMD, mgHA/cm^3^	539	579	802	854	263.6 (+48.9 %)	274.8 (+47.4 %)
Tb.Th, mm	0.054	0.079	0.059	0.100	0.005 (+7.8 %)	0.021 (+27.1 %)
Ct.Th, mm	0.14	0.15	0.81	1.01	0.67 (+464 %)	0.86 (+569 %)


DXA	BMD, g/cm^2^	*Z*-score	BMD, g/cm^2^	Z-score	Variation⁎⁎
Lumbar spine (L1-L4)	0.786	−1.8	1.116	−0.8	0.330 (+41.9 %)
Total body less head	0.628	−2.5	0.827	−1.5	0.199 (+31.6 %)

DXA: dual X-ray absorptiometry. HR-pQCT: high resolution peripheral quantitative computed tomography. BMD: bone mineral density. Tt.vBMD: total volumetric bone mineral density; Tb.vBMD: trabecular volumetric bone mineral density; Ct.vBMD: cortical volumetric bone mineral density; Tb.Th: trabecular thickness; Ct.Th: cortical thickness.

⁎ HR-pQCT coefficient of variation: 0.93–1.41 % at the distal radius and 0.25–1.16 % at the distal tibia for density measurements, and 1.49–7.59 % at the distal radius and 0.78–6.35 % at the distal tibia for morphometric measurements.

⁎⁎ Least significant variation: lumbar spine 0.033 g/cm^2^; total body less head 0.016 g/cm^2^.

**Fig. 2:**
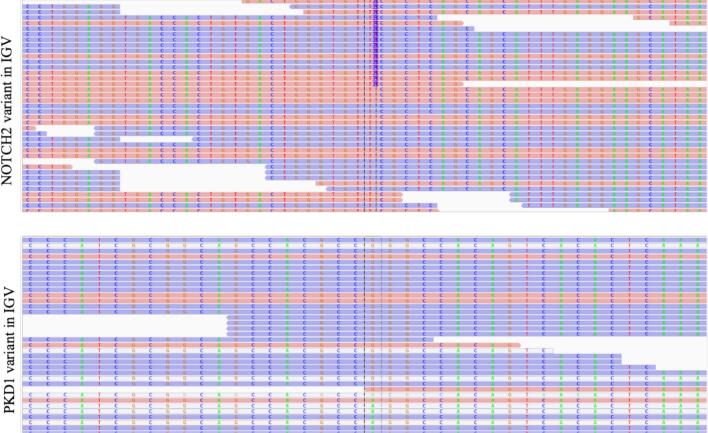
Visualization of the *NOTCH2* (upper) and *PKD1* (lower) variant in IGV. *NOTCH2* (upper): Alignment of sequencing reads mapped to the *NOTCH2* gene (GRCh38: NC_000001.11:g.119915522_119915523insTG, NM_024408.3:c.7199_7200insTG, p.Thr2401Glufs*16) is highlighted between the dotted vertical lines. Reads are colored by strand orientation (blue and salmon). The frameshift insertion (T > TCA) is represented by the number 2, indicating the presence of an insertion event detected in multiple reads. The high coverage at this position supports the confidence of the variant call. *PKD1* (lower): Alignment of sequencing reads mapped to the *PKD1* gene (GRCh38: NC_000016.10:g.2118807 A > G, NM_001009944.3:c.398 T > C, p.Leu133Pro) is highlighted between the dotted vertical lines. Reads are colored by strand orientation (blue and salmon). The heterozygous c.398 T > C substitution is visible in multiple reads. Coverage depth is adequate at the variant position, and no alignment artifacts are observed.IGV: Integrated Genomics Viewer (Broad Institute, Cambridge, MA, USA).

Due to the low BMD and significant impairment of bone microarchitecture in a young patient with no apparent secondary causes, genetic testing (whole exome sequencing) was conducted, revealing a causative variant in the *NOTCH2* (neurogenic locus notch homolog protein 2) gene (*NOTCH2*, (GRCh38): NC_000001.11: g.119915522_119915523insTG, NM_024408.3: c.7199_7200insTG, p.Thr2401Glufs*16). The genetic variant was absent in his parents and is classified as pathogenic based on the ACMG (American College of Medical Genetics and Genomics) criteria, fulfilling PVS1 (loss of function), PS2 (de novo mutation), PM2 (absence in population databases), and PP4 (phenotypic specificity) (Richards et al., 2015). This finding confirmed the diagnosis of Hajdu-Cheney syndrome (HCS). Interestingly, a potentially pathogenic variant was identified in *PKD1* (polycystic kidney disease 1) gene (*PKD1*, (GRCh38): NC_000016.10: g.2118807 A > G, NM_001009944.3:c.398 T > C, p.Leu133Pro), the gene most frequently mutated in ADPKD. Whole-exome sequencing was performed with an average coverage of 71×, with 99.5 % of targeted bases covered at ≥10×. The *NOTCH2* and *PKD1* variants were called with high confidence, with quality scores of 1385.73 (depth: 112×) and 455.77 (depth: 62×), respectively. These metrics are illustrated in the IGV (Integrative Genomics Viewer) figures (Fig. 2). If indeed located in *PKD1*, this second variant is positioned in the gene region that duplicates into six pseudogenes proximally on chromosome 16. Assumed as in *PKD1*, this variant is classified as likely pathogenic according to the ACMG criteria, fulfilling PM2 (absence in population databases), PP1 (segregation with ADPKD in the family), and PP4 (a phenotype specific to the disease). The mentioned pseudogenes, however, share very high sequence identity with the duplicated region of *PKD1* (Hughes et al., 1995), a particularity that in principle complicates the assignment of the detected variant as causative. However, the facts that the patient's father is affected by ADPKD and harbors the same variant provided support to the patient's diagnosis of ADPKD.

Interestingly, our patient also harbored a likely causative missense variant in *PKD1*, in this case inherited from his father. It must be noted that this variant could not be definitely confirmed as a pathogenic variant in *PKD1* due to limitations of genetic testing based on short reads. This method lacks specificity for the 5′ portion of *PKD1*, since the related pseudogenes share up to 98 % sequence identity with exons 1–32 of *PKD1* (Ali et al., 2019). However, since the patient's father has an established diagnosis of ADPKD and is a carrier of the same variant, it is highly likely that the mentioned variant is indeed pathogenic and, therefore, the primary cause of the patient's polycystic kidney phenotype.

The treatment plan for the established osteoporosis included weekly alendronate (70 mg orally, once per week), along with impact exercise (muscle strengthening and gymnastics, under the guidance of a physical therapist and respecting the patient's preferences), a calcium-rich diet (1200 mg/day), and vitamin D supplementation aimed at maintaining serum levels of 25-OH vitamin D above 30 ng/mL.

After 3 years of treatment, follow-up DXA scans showed improvement in BMD (Table 1) and HR-pQCT revealed remarkable improvement in vBMD, especially in the cortical bone (Table 1 and Fig. 1). At this point, a decision was made to suspend alendronate (drug holiday) with continued annual monitoring of BMD using DXA and HR-pQCT to assess long-term outcomes. The patient maintained a calcium-rich diet (targeting 1200 mg/day), vitamin D supplementation to keep serum levels above 30 ng/mL, and a regular exercise regimen (including muscle strengthening) to support bone health.

## 3 Discussion

We report the case of a young patient with Hajdu-Cheney Syndrome (HCS) associated with autosomal dominant polycystic kidney disease (ADPKD), both diagnosed clinically and genetically. This patient presented with systemic bone involvement, established osteoporosis, and polycystic kidneys. Genetic testing was prompted by the patient's youth, absence of a family history of osteoporosis, occurrence of a fragility fracture, and significant bone impairment without apparent secondary causes. He also displayed a polycystic kidney phenotype and a family history of ADPKD.

HCS is an autosomal dominant skeletal disorder characterized by facial anomalies, acro-osteolysis, generalized osteoporosis, short stature, insufficient ossification of the skull, and periodontal disease (Hajdu and Kauntze, 1948; Brennan and Pauli, 2001). Despite its autosomal dominant inheritance, several sporadic cases likely caused by de novo variants have been reported (Majewski et al., 2011). The *NOTCH2* variant identified in the current case (p.Thr2401GluFS*16) affects the PEST (proline, glutamate, serine, and threonine) domain. This domain is involved in protein degradation, typically mediating proteasomal degradation (Isidor et al., 2011). The truncating p.Thr2401GluFS*16 variant, therefore, leads to a gain-of-function effect on NOTCH2 protein, and classically associates with HCS (Zhao et al., 2013). Of note, loss of this domain prolongs the activity of Notch intracellular domain (NICD), resulting in excessive signaling (Simpson et al., 2011; Zhao et al., 2013). Animal models targeting *Notch2* exhibit smaller body size, reduced bone mineral density in both cortical and trabecular bone, increased osteoclastogenesis, and increased bone resorption (Canalis et al., 2016). The role of this gene in osteoclastogenesis has also been demonstrated in other studies (Canalis et al., 2024). Conversely, the use of anti-Notch2 antibodies in animal models prevented or reversed bone loss (Canalis et al., 2017). Our case represents the first long-term follow-up of bone mass using both DXA and HR-pQCT in a monogenic disease associated with a de novo variant and early-onset osteoporosis.

Interestingly, our patient also harbored a likely causative missense variant in *PKD1*, in this case inherited from his father. It must be noted that this variant could not be definitely confirmed as a pathogenic variant in *PKD1* due to limitations of genetic testing based on short reads. This method lacks specificity for the 5′ portion of *PKD1*, since the related pseudogenes share up to 98 % sequence identity with exons 1–32 of *PKD1 (*Ali et al., *2019**)*). However, since the patient's father has a well-established diagnosis of ADPKD and is a carrier of the same variant, it is highly likely that the mentioned variant is indeed pathogenic and, therefore, the primary cause of the patient's polycystic kidney phenotype.

The implications of *PKD1* variants in bone metabolism are potentially relevant. Polycystins-1 and -2 (PC1 and PC2), the protein products of *PKD1* and *PKD2*, respectively, are known to play critical roles in bone metabolism. PC1 acts as a mechanosensor in osteoblasts, affecting bone formation and mineral density (Xiao and Quarles, 2010). Disruption of *Pkd1* in osteoblasts has been shown to reduce bone mineral density, cortical thickness, and trabecular bone volume in mice, contributing to osteopenia and impaired bone formation (Li et al., 2017; Lu et al., 2001). Moreover, patients with ADPKD often exhibit elevated levels of fibroblast growth factor 23 (FGF23), a growth factor known to influence phosphate metabolism. Increased FGF23 levels are associated with impaired phosphate handling and exacerbated bone mineral disease (Pavik et al., 2011). In our patient, these mechanisms may have partially contributed to the development of the observed bone abnormalities, particularly because of the link between *PKD1* pathogenic variants and impaired bone formation.

Our patient was treated with alendronate, a bisphosphonate, which has been shown to inhibit osteoclast-mediated bone resorption (Drake et al., 2008; Khosla et al., 2012). This treatment aligns with the known pathogenesis of HCS, where increased osteoclast activity is a key factor in bone degradation. Bisphosphonates, including alendronate, are known to bind to hydroxyapatite in bone, inhibiting osteoclast attachment and reducing bone resorption (Drake et al., 2008). Bisphosphonates have also been reported to improve bone mineral density (BMD) in some cases of HCS, while other studies have failed to show a clear benefit (Pittaway et al., 2018; Ahmad et al., 2021). Despite the uncertainty regarding the efficacy of bisphosphonates in HCS patients, our patient exhibited significant improvements in BMD and bone microarchitecture after three years of treatment, particularly in the cortical bone, which is crucial for structural rigidity. There was an apparent, though very subtle, reduction in trabecular bone. However, a study has shown that HCS patients have greater trabecular bone volume than healthy controls, suggesting that the treatment may not have significantly altered this parameter, which is naturally increased (Sakka et al., 2017).

With increased access to genetic diagnosis, more cases of HCS have been described in recent years. This is particularly important for rheumatologists, as such patients present with acro-osteolysis and calcinosis, sometimes accompanied by periungual capillaroscopy changes and thus constituting a differential diagnosis for systemic sclerosis (Botou et al., 2017; Damian et al., 2016). Antiresorptive therapy remains largely empirical and associated with inconsistent effects (Drake et al., 2008; Khosla et al., 2012). It should be noted that bisphosphonates may potentially prevent initiation of bone formation in humans and may not allow activation of bone remodeling (Jensen et al., 2021). With the focus on acro-osteolysis and BMD by densitometry in HCS cases, a previous study found no impact on localized bone, while improvement in densitometry *Z*-scores was noted (Pittaway et al., 2018). In addition, Ahmad et al., 2021 (Ahmad et al., 2021), reported significant improvement in a HCS patient's BMD after two years of treatment with zoledronic acid. Other case reports included the use of denosumab in an adult patient with previous strontium ranelate bisphosphonate use (Adami et al., 2016) and the use of romosozumab (Kim et al., 2023). While the improvement in our patient's bone health could theoretically be attributed to other factors, such as increased calcium intake, physical exercise, or vitamin D supplementation, these factors are less likely to explain the observed substantial BMD and microarchitectural gains. Indeed, the magnitude of his response suggests that alendronate was the primary driver of improvement. The patient's young age at the start of treatment and the progressive nature of HCS also make it unlikely that bone density would have improved spontaneously or only due to lifestyle factors (Ahmad et al., 2021; Sakka et al., 2017).

The molecular mechanism through which bisphosphonates exert their effects in patients with HCS remains speculative, but it likely involves the inhibition of osteoclast activity mediated by NOTCH2 overactivation (Canalis et al., 2016). By inhibiting osteoclasts, bisphosphonates such as alendronate reduce bone resorption, thereby counteracting the increased bone turnover seen in HCS. Given the role of NOTCH2 in osteoclastogenesis, bisphosphonate therapy may help rebalance the dysregulated bone remodeling process (Drake et al., 2008; Sakka et al., 2017).

In conclusion, our patient showed a remarkable response to alendronate, with significant improvements in BMD and bone microarchitecture and without new fragility fractures. Therefore, early recognition and treatment of primary causes of osteoporosis, such as HCS, are essential to prevent the morbidity associated with bone fractures.

## CRediT authorship contribution statement

**André Silva Franco:** Writing – review & editing, Writing – original draft, Supervision, Investigation, Formal analysis, Conceptualization. **Valeria de Falco Caparbo:** Writing – review & editing, Project administration, Data curation. **Elieser Hitoshi Watanabe:** Writing – review & editing, Writing – original draft, Investigation. **Rosa Maria Rodrigues Pereira:** Supervision, Investigation, Conceptualization. **Luiz Fernando Onuchic:** Writing – review & editing, Writing – original draft, Supervision, Investigation, Conceptualization.

## Ethical form

The authors declare that they have obtained written and signed consent to publish the case report from the patient and their legal guardians.

## Declaration of competing interest

The authors declare that they have no known competing financial interests or personal relationships that could have appeared to influence the work reported in this paper.

## Data Availability

Data will be made available on request.

## References

[bb0005] Adami G., Rossini M., Gatti D., Orsolini G., Idolazzi L., Viapiana O., Scarpa A., Canalis E. (2016). Hajdu Cheney syndrome; report of a novel NOTCH2 mutation and treatment with denosumab. Bone.

[bb0010] Ahmad A., Deeb H., Alasmar D. (2021). Hajdu Cheney syndrome; a novel NOTCH2 mutation in a Syrian child, and treatment with zolidronic acid: a case report and a literature review of treatments. Ann Med Surg (Lond)..

[bb0015] Ali H., Hussain S., Akhtar T., Bhanji R.A., Naeem M. (2019). PKD1 duplicated regions limit clinical utility of whole exome sequencing for genetic diagnosis of autosomal dominant polycystic kidney disease. Sci. Rep..

[bb0020] Botou A., Bangeas A., Alexiou I., Sakkas L.I. (2017). Acro-osteolysis. Clin. Rheumatol..

[bb0025] Brennan A.M., Pauli R.M. (2001). Hajdu--Cheney syndrome: evolution of phenotype and clinical problems. Am. J. Med. Genet..

[bb0030] Canalis E., Schilling L., Yee S.P., Lee S.K., Zanotti S. (2016). Hajdu Cheney mouse mutants exhibit osteopenia, increased Osteoclastogenesis, and bone resorption. J. Biol. Chem..

[bb0035] Canalis E., Sanjay A., Yu J., Zanotti S. (2017). An antibody to Notch2 reverses the Osteopenic phenotype of Hajdu-Cheney mutant male mice. Endocrinology.

[bb0040] Canalis E., Schilling L., Yu J., Denker E. (2024). NOTCH2 promotes osteoclast maturation and metabolism and modulates the transcriptome profile during osteoclastogenesis. J. Biol. Chem..

[bb0045] Costantini A., Mäkitie R.E., Hartmann M.A., Fratzl-Zelman N., Zillikens M.C., Kornak U., Søe K., Mäkitie O. (2022). Early-onset osteoporosis: rare monogenic forms elucidate the complexity of disease pathogenesis beyond type I collagen. J. Bone Miner. Res..

[bb0050] Damian L.O., Simon S.P., Filipescu I., Bocsa C., Botar-Jid C., Rednic S. (2016). Capillaroscopic findings in a case of Hajdu-Cheney syndrome. Osteoporos. Int..

[bb0055] Drake M.T., Clarke B.L., Khosla S. (2008). Bisphosphonates: mechanism of action and role in clinical practice. Mayo Clin. Proc..

[bb0060] Formosa M.M., Christou M.A., Mäkitie O. (2024). Bone fragility and osteoporosis in children and young adults. J. Endocrinol. Investig..

[bb0065] Hajdu N., Kauntze R. (1948). Cranio-skeletal dysplasia. Br. J. Radiol..

[bb0070] Hughes J., Ward C.J., Peral B., Aspinwall R., Clark K., San Millán J.L., Gamble V., Harris P.C. (1995). The polycystic kidney disease 1 (PKD1) gene encodes a novel protein with multiple cell recognition domains. Nat. Genet..

[bb0075] Isidor B., Lindenbaum P., Pichon O., Bézieau S., Dina C., Jacquemont S., Martin-Coignard D., Thauvin-Robinet C., Le Merrer M., Mandel J.L., David A., Faivre L., Cormier-Daire V., Redon R., Le Caignec C. (2011). Truncating mutations in the last exon of NOTCH2 cause a rare skeletal disorder with osteoporosis. Nat. Genet..

[bb0080] Jensen P.R., Andersen T.L., Chavassieux P., Roux J.P., Delaisse J.M. (2021). Bisphosphonates impair the onset of bone formation at remodeling sites. Bone.

[bb0085] Khosla S., Bilezikian J.P., Dempster D.W., Lewiecki E.M., Miller P.D., Neer R.M., Recker R.R., Shane E., Shoback D., Potts J.T. (2012). Benefits and risks of bisphosphonate therapy for osteoporosis. J. Clin. Endocrinol. Metab..

[bb0090] Kim K.J., Hong N., Lee S., Shin S., Rhee Y. (2023). Exploratory use of romosozumab for osteoporosis in a patient with Hajdu-Cheney syndrome: a case report. Osteoporos. Int..

[bb0095] Li S, Xu W, Xing Z, Qian J, Chen L, Gu R, Guo W, Lai X, Zhao W, Li S, Wang Y, Wang QJ, Deng F. A conditional knockout mouse model reveals a critical role of PKD1 in osteoblast differentiation and bone development. Sci. Rep. 2017;7:40505. doi:10.1038/srep40505. PMC5233966 28084409

[bb0100] Lu W., Shen X., Pavlova A., Lakkis M., Ward C.J., Pritchard L., Harris P.C., Genest D.R., Perez-Atayde A.R., Zhou J. (2001). Comparison of Pkd1-targeted mutants reveals that loss of polycystin-1 causes cystogenesis and bone defects. Hum. Mol. Genet..

[bb0105] Majewski J., Schwartzentruber J.A., Caqueret A., Patry L., Marcadier J., Fryns J.P., Boycott K.M., Ste-Marie L.G., FE McKiernan, Marik I., Van Esch H., FORGE Canada Consortium, Michaud J.L., Samuels M.E. (2011). Mutations in NOTCH2 in families with Hajdu-Cheney syndrome. Hum. Mutat..

[bb0110] Pavik I., Jaeger P., Kistler A.D., Poster D., Krauer F., Cavelti-Weder C., Rentsch K.M., Wüthrich R.P., Serra A.L. (2011). Patients with autosomal dominant polycystic kidney disease have elevated fibroblast growth factor 23 levels and a renal leak of phosphate. Kidney Int..

[bb0115] Pittaway JFH, Harrison C, Rhee Y, Holder-Espinasse M, Fryer AE, Cundy T, Drake WM, Irving MD. Bisphosphonate therapy for spinal osteoporosis in Hajdu-Cheney syndrome - new data and literature review. Orphanet J Rare Dis. 2018Apr 4;13(1):47. doi:10.1186/s13023-018-0795-5. PMC5885380 29618366

[bb0120] Richards S., Aziz N., Bale S., Bick D., Das S., Gastier-Foster J., Grody W.W., Hegde M., Lyon E., Spector E., Voelkerding K., Rehm H.L. (2015). Standards and guidelines for the interpretation of sequence variants: a joint consensus recommendation of the American College of Medical Genetics and Genomics and the Association for Molecular Pathology. Genet. Med..

[bb0125] Sakka S., Gafni R.I., Davies J.H., Clarke B., Tebben P., Samuels M., Saraff V., Klaushofer K., Fratzl-Zelman N., Roschger P., Rauch F., Högler W. (2017). Bone structural characteristics and response to bisphosphonate treatment in children with Hajdu-Cheney syndrome. J. Clin. Endocrinol. Metab..

[bb0130] Sakka SD. Osteoporosis in children and young adults. Best Pract. Res. Clin. Rheumatol. 2022 Sep;36(3):101776. doi:10.1016/j.berh.2022.101776. 36109301

[bb0135] Simpson M.A., Irving M.D., Asilmaz E., Gray M.J., Dafou D., Elmslie F.V., Mansour S., Holder S.E., Brain C.E., Burton B.K., Kim K.H., Pauli R.M., Aftimos S., Stewart H., Kim C.A., Holder-Espinasse M., Robertson S.P., Drake W.M., Trembath R.C. (2011). Mutations in NOTCH2 cause Hajdu-Cheney syndrome, a disorder of severe and progressive bone loss. Nat. Genet..

[bb0140] Veronese N., Briot K., Guañabens N., Albergaria B.H., Alokail M., Al-Daghri N., Bemden A.B., Bruyère O., Burlet N., Cooper C., Curtis E.M., Ebeling P.R., Halbout P., Hesse E., Hiligsmann M., Camargos B.M., Harvey N.C., Perez A.D., Radermecker R.P., Reginster J.Y., Rizzoli R., Siggelkow H., Cortet B., Brandi M.L. (2024). Recommendations for the optimal use of bone forming agents in osteoporosis. Aging Clin. Exp. Res..

[bb0145] Xiao Z.S., Quarles L.D. (2010). Role of the polycytin-primary cilia complex in bone development and mechanosensing. Ann. N. Y. Acad. Sci..

[bb0150] Zhao W., Petit E., Gafni R.I., Collins M.T., Robey P.G., Seton M., Miller K.K., Mannstadt M. (2013). Mutations in NOTCH2 in patients with Hajdu-Cheney syndrome. Osteoporos. Int..

